# The magnitude and durability of neutralizing antibody responses to human papillomavirus vaccine do not depend on DNA sensing pathways

**DOI:** 10.1128/jvi.02150-25

**Published:** 2026-02-10

**Authors:** Juhi Patel, Zida Yang, Sara Ping, Alaa Ahmed, Trevor W. Simon, Jesse J. Waggoner, Erin M. Scherer

**Affiliations:** 1 Division of Infectious Diseases, Department of Medicine, Emory University School of Medicine 12239, Atlanta, Georgia, USA; 2 Department of Pathology and Laboratory Medicine, Emory University School of Medicine 12239, Atlanta, Georgia, USA; Tufts University School of Medicine, Boston, Massachusetts, USA

**Keywords:** human papillomavirus, vaccine, neutralizing antibody, plasma cell, DNA

## Abstract

How human papillomavirus (HPV) vaccines elicit robust and enduring neutralizing antibody (nAb) responses is unknown, yet such information is valuable to vaccinology. In addition to the major capsid protein, L1, the 4-valent HPV vaccine reportedly also contains recombinant L1 DNA. Nucleic acids in vaccines enhance antibody responses and are employed as adjuvants, but whether L1 DNA enhances HPV vaccine antibody responses is understudied. We tested whether 9-valent HPV (9vHPV) vaccine lots contained L1 DNA and if peak or long-term 9vHPV vaccine-elicited nAb responses were dependent on DNA sensors Toll-like receptor 9 (TLR9), AIM2, or cGAS or on STING or MyD88, the adaptors for cGAS and TLR9. To quantify L1 DNA, we extracted total nucleic acid per 9vHPV dose and applied quantitative PCR to amplify HPV6/11/16/18/31/33/45/52/58 L1 sequences. Wild-type mice (C57BL/6J) or mice deficient in DNA sensing pathways (TLR9^-/-^, AIM2^-/-^, cGAS^-/-^, STING^gt/gt^, MyD88^-/-^) were administered 9vHPV vaccine or equivalent adjuvant only at 0, 4, and 12 weeks. We measured serum HPV16 and HPV18 nAb titers at 16, 26, 40, and 53–56 weeks and HPV16- and HPV18-specific bone marrow plasma cell responses at 53–56 weeks. We detected 5–44 and 104–351 copies of HPV6 and HPV18 L1 DNA per 9vHPV dose, but no other types (*n* = 3 lots). We found no consistent difference in geometric mean nAb titers between mice strains that received 9vHPV at any time point tested or in median HPV-specific plasma cell frequencies. Thus, we conclude the magnitude and durability of nAb responses to 9vHPV vaccination do not depend on DNA sensing pathways.

**IMPORTANCE:**

How the highly effective human papillomavirus (HPV) vaccines elicit strong and long-lasting antibody responses is unknown, yet such knowledge is valuable to vaccine development. The HPV vaccines are comprised of virus-like particles assembled from the HPV major capsid protein, L1; an adjuvant; and reportedly also residual recombinant L1 DNA as a result of the manufacturing process. We tested whether the currently available 9-valent HPV (9vHPV) vaccine contained L1 DNA and if 9vHPV vaccine-elicited antibody responses were dependent on DNA sensing pathways of the innate immune system *in vivo*. We confirmed separate lots of the 9vHPV vaccine contained low copy numbers of L1 DNA, but found 9vHPV vaccine-generated antibody responses were not dependent on DNA sensing. This study has thus refined our understanding of potential mechanisms underlying the strong and long-lasting antibody responses to HPV vaccination.

## INTRODUCTION

Antibodies play a key role in vaccine-induced prevention of infectious diseases (1, 2). Because live attenuated vaccines may not be feasible for all pathogens or recipients, the development of subunit vaccines that elicit potent and durable antibody responses is essential. However, knowledge of how to reliably design subunit vaccines that elicit long-lived antibody responses is lacking.

Human papillomavirus (HPV) vaccines are examples of subunit vaccines that induce robust humoral immunity for many years with extremely slow antibody decay rates (3–8). HPV vaccine-induced antibody durability is superior to that of many other approved inactivated and subunit vaccines (8–17). Thus, HPV vaccines represent a model for studying how lasting subunit vaccine humoral immunity is generated. The 9-valent HPV (9vHPV) vaccine is the only licensed HPV vaccine currently available in the USA. It protects against HPV types that commonly cause genital warts (HPV 6 and 11) and cancer (HPV 16, 18, 31, 33, 45, 52, and 58) (18–22). HPV vaccines are comprised of virus-like particles (VLPs), where each HPV type VLP assembles from 360 units of its major capsid protein, L1 (23–25). During HPV infection, L1 binds to and packages the 8 kb double-stranded (ds) HPV DNA genome into virions (26, 27). HPV vaccines do not contain the HPV genome, and L1 is a structural protein, not an oncogene/oncoprotein (28, 29). That is, L1 cannot cause HPV-associated cancers like the E6 and E7 proteins of high-risk HPV types can (28, 29). L1 is also capable of binding and packaging other dsDNA, like recombinant DNA plasmids (30). Thus, it is perhaps unsurprising that the US Food and Drug Administration (US FDA) reported that the 4-valent HPV vaccine, the predecessor of the 9vHPV vaccine, contains residual recombinant L1 DNA as a result of the manufacturing process, which poses no safety risk (31).

Foreign DNA in humans is sensed by various cellular pattern recognition receptors (PRRs), such as Toll-like receptor 9 (TLR9), which senses hypomethylated DNA CpG sites in the endosomal compartment, and cyclic GMP-AMP synthase (cGAS) and absent in melanoma 2 (AIM2), which sense dsDNA in the cytosol (32, 33). Upon recognizing foreign DNA, these PRRs activate pathways that result in the secretion of pro-inflammatory cytokines and type I interferons, which in turn boost antigen presentation (32, 33).

Antigen-associated DNA-based ligands have been investigated and approved as adjuvants because they enhance antibody, germinal center B cell, and plasmablast responses in animal models and humans (34–39). For example, the TLR9 agonist-based adjuvant in the Helisav-B hepatitis B virus (HBV) vaccine, CpG 1018, induces improved seroprotection rates compared to alum adjuvants of other HBV vaccines (38, 39). TLR9 agonists have also been shown to improve antibody and/or B cell responses to HPV L1 pentamers (40), haptenated protein antigens (36), and West Nile virus envelope (37) compared to antigen alone or antigen and alum. In addition, nucleotide molecules like those produced from dsDNA by cGAS, which activate stimulator of interferon genes (STING), have been shown to improve antibody and B cell responses to HIV gp41 protein, including antibody response durability, relative to antigen alone or antigen and monophosphoryl lipid A (34). Indeed, as little as 200 ng of STING agonists enhance antibody and B cell responses to influenza hemagglutinin protein, including protection against influenza challenge in mice, compared to antigen alone or antigen and alum (35).

HPV 16 and 18 L1 open reading frames have several CpG sites (41). However, whether HPV vaccine-elicited antibody responses depend on DNA sensing pathways has not been studied to our knowledge. Thus, we tested whether we could detect HPV L1 DNA in 9vHPV vaccine lots and, if so, whether peak and long-lived 9vHPV vaccine-elicited antibody responses depended on DNA sensing pathways.

## RESULTS

### Measuring HPV L1 DNA levels in different 9vHPV vaccine lots

We first sought to confirm whether HPV L1 DNA could be detected in 9vHPV vaccine. To do this, nucleic acids were extracted from single doses (500 µL each) of three separate 9vHPV vaccine lots. Individual 9vHPV doses were divided in half (250 µL × 2) prior to extraction to generate technical replicates, and singleplex quantitative PCR reactions were performed on each replicate to amplify HPV 6, 11, 16, 18, 31, 45, 52, or 58 L1 DNA with primers/probes targeting naturally occurring HPV L1 sequences (42). As shown in Table 1, we reproducibly detected HPV 6 and 18 L1 DNA in each of three 9vHPV lots at estimated copy numbers of 25–44 or 104–351 per 500 µL 9vHPV dose, respectively. Other types were not detected. This confirmed that HPV L1 DNA was present in 9vHPV vaccine. We also attempted unbiased sequencing from PCR-positive DNA eluates from lots 2 and 3 as an alternate approach for detecting other HPV L1 types in the vaccine. However, no HPV DNA was detected in either experiment by deep sequencing (data not shown); this is unsurprising given the extremely low concentrations of HPV DNA detected by PCR. In addition, because non-HPV DNA in the vaccine could also activate DNA sensing pathways, we also quantified the total DNA in eluates from lots 2 and 3 using the Qubit dsDNA High Sensitivity Assay. However, both samples yielded fluorescence signals that were below the assay’s detection limit (0.1 ng).

**TABLE 1:** Magnitude of HPV L1 DNA detected in 9vHPV vaccine lots

	9vHPV vaccine lot 1	9vHPV vaccine lot 2	9vHPV vaccine lot 3	Median
HPV6 L1 DNA	25^*a*^	5	44	25
HPV18 L1 DNA	104	351	191	191
All other 9vHPV-type L1 DNA	ND^*b*^	ND	ND	

^*a*^ Copies/9vHPV vaccine dose.

^*b*^ ND, not detected.

### Determining a 9vHPV vaccine dose for the mouse model

**Fig 1:**
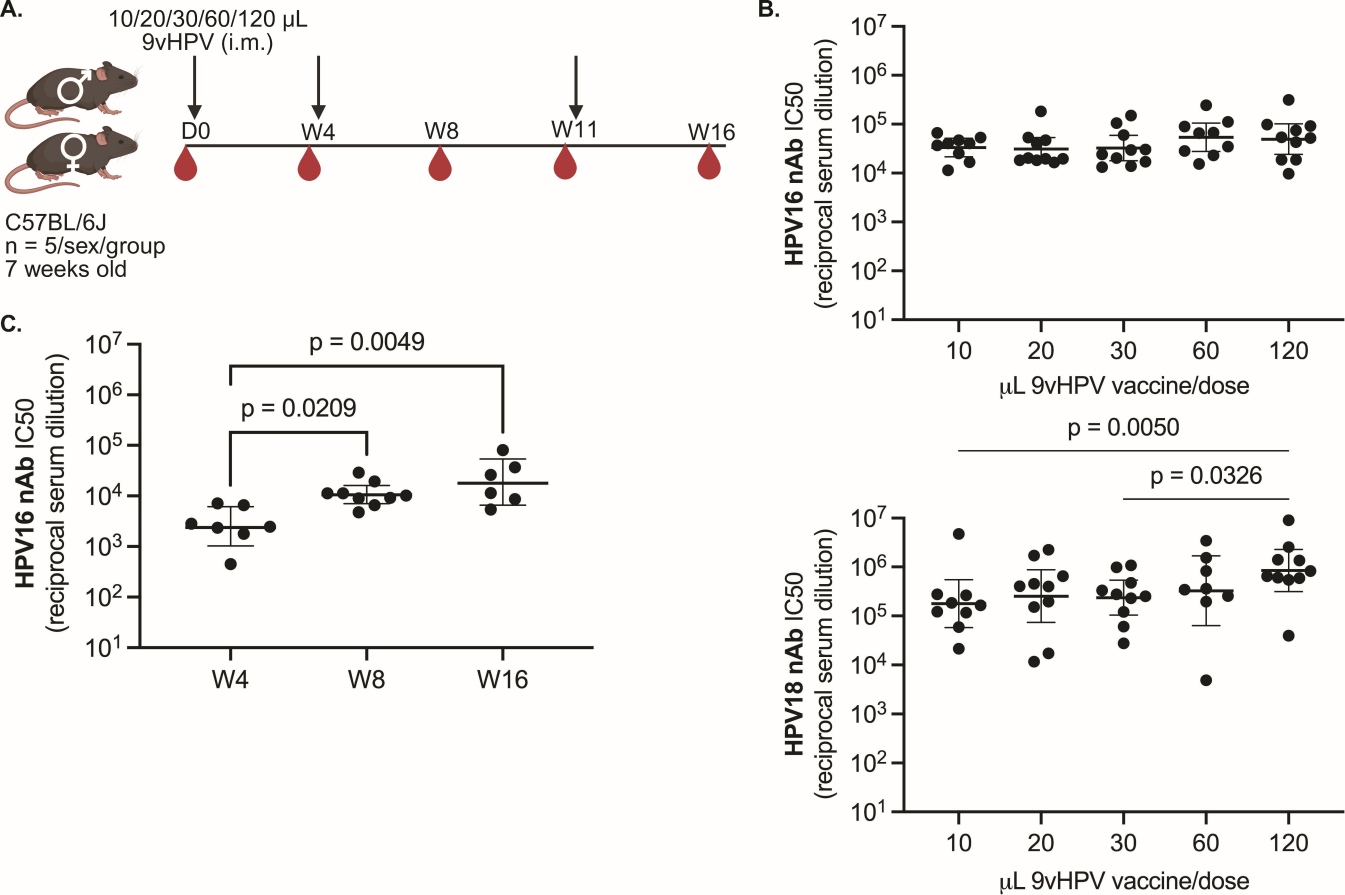
9vHPV vaccine dose titration to identify a dose volume for mice. (**A**) Study schematic (D, study day; W, study week; i.m., intramuscularly) created with BioRender. (**B**) Peak serum HPV16 and HPV18 nAb titers approximately 1 month after the third and final dose at week 16 in mice (*n* = 8–10 per group) that received the indicated dose volumes; 60 μL was selected as the lowest volume that elicited non-inferior HPV16 and HPV18 nAb responses at week 16. (**C**) Serum HPV16 nAb titers approximately 1 month after the first, second, or third 60 μL 9vHPV vaccine dose (*n* = 6–9 mice per time point). Geometric mean with 95% confidence intervals shown, where each point represents the result from an individual mouse; Kruskal-Wallis with Dunn’s post-test between dose groups.

Prior to testing whether peak and long-lived 9vHPV vaccine-elicited antibody responses depended on DNA sensing pathways in the mouse model, we first needed to select a vaccine dose for mice. We tested different dose volumes of 9vHPV vaccine in five groups of male (*n* = 5) and female (*n* = 5) wild-type (wt; C57BL/6J) mice: approximately 1/4 (120 μL), 1/8 (60 μL), 1/16 (30 μL), 1/32 (20 μL), and 1/64 (10 μL) of the human dose volume (Fig. 1A). According to the 9vHPV vaccine prescribing information, this corresponds to 4.8–14.4 μg HPV L1 protein for the highest dose or 0.4–1.2 μg HPV L1 protein for the lowest dose, exact amount dependent on HPV type. These 9vHPV dose volumes were administered intramuscularly (i.m.) at day 0, week 4, and week 11 in each study group (Fig. 1A) to mimic the adult human 9vHPV dose schedule that is given at 0, 2, and 6 months. As shown in Fig. 1B, when HPV16 neutralizing antibody (nAb) titers were assessed in sera collected approximately 1 month after the final dose at week 16 by pseudovirus (psV) neutralization assay, there was no difference in titers by dose volume. However, both the 10 µL and 30 µL dose volumes generated significantly lower HPV18 nAb titers compared to the highest dose volume. Thus, we selected the 60 µL 9vHPV dose volume as the lowest 9vHPV dose volume that did not elicit inferior nAb responses at week 16. As shown in Fig. 1C, HPV16 nAb titers peaked approximately 1 month after the second 60 µL 9vHPV dose at week 8 and stayed elevated approximately 1 month after the final 60 µL 9vHPV dose at week 16. Moreover, male and female mice generated similar HPV nAb titers to the 9vHPV vaccine (Fig. S1).

### Testing whether peak 9vHPV vaccine-elicited antibody responses depend on DNA sensing pathways

**Fig 2:**
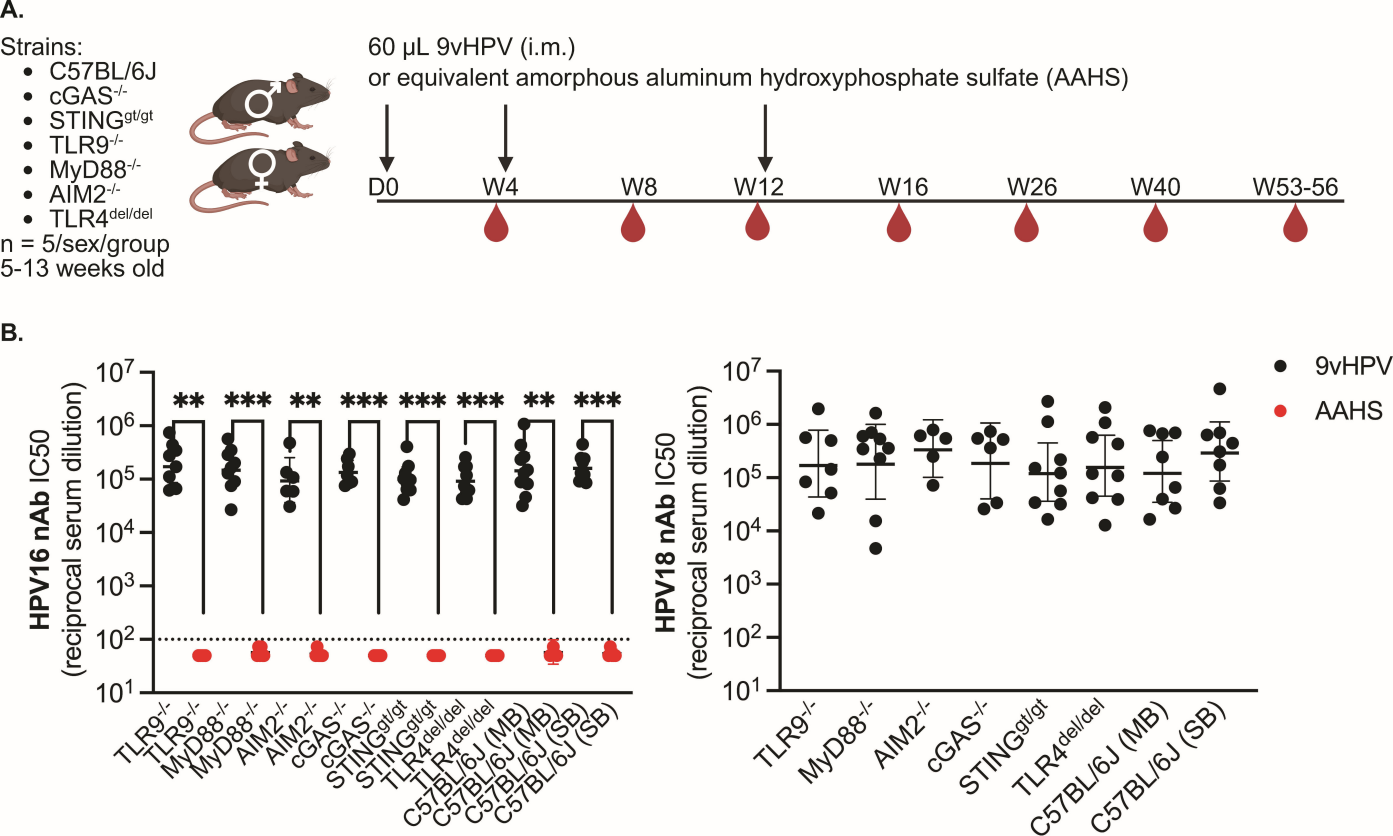
The magnitude of peak nAb responses to the 9vHPV vaccine does not depend on DNA sensing pathways. (**A**) Study schematic created with BioRender. (**B**) Peak serum HPV16 or HPV18 nAb titers approximately 1 month after the third and final dose at week 16 in mice that received 9vHPV (*n* = 5–9 per group), which contains AAHS as an adjuvant, or equivalent AAHS (60 μg) only (*n* = 3–8 per group). MB or SB represents maximum barrier or standard barrier vivarium rooms where mice were housed. Geometric mean with 95% confidence intervals shown, where each point represents the result from an individual mouse; Kruskal-Wallis with Dunn’s post-test comparing similarly housed strains to control (wild-type C57BL/6J mice); Mann-Whitney U-test between 9vHPV and AAHS groups for a given strain (**P = 0.0022–0.007; ***P = 0.0002–0.001). Dashed line represents cutoff for neutralizing activity; results below this line were non-neutralizing.

To test whether peak 9vHPV vaccine-elicited antibody responses depend on DNA sensing pathways, we administered three, 60 µL 9vHPV doses at day 0, week 4, and week 12 in groups of wild-type mice (male *n* = 5; female *n* = 5), as well as mice globally deficient in TLR9, cGAS, and AIM2 DNA sensing pathways (TLR9^-/-^, cGAS^-/-^ [43], AIM2^-/-^ [44]; Fig. 2A). We included mice globally deficient in myeloid differentiation factor 88 (MyD88) and STING as controls (MyD88^-/-^ [45] and STING^gt/gt^ [46]; Fig. 2A), because TLR9 and cGAS signal through these adaptors, respectively. We also included mice globally deficient in TLR4 (TLR4^del/del^ [47, 48]; Fig. 2A), as prior work has reported conflicting results on the importance of this PRR pathway to antibody responses elicited by HPV VLPs in mice (49, 50). In addition to 9vHPV, we also administered three doses of equivalent adjuvant only as a negative control (amorphous aluminum hydroxyphosphate sulfate, AAHS; 60 µg) at day 0, week 4, and week 12 to separate groups of the same strains of mice (Fig. 2A). Serum from mice that received AAHS did not exhibit HPV neutralizing activity 1 month post-third/final dose at week 16 as expected, given that these mice received no HPV L1 antigen (Fig. 2B), whereas serum from mice that received 9vHPV exhibited potent HPV16 and HPV18 neutralizing activity (Fig. 2B). However, no differences in HPV16 or HPV18 nAb titers were observed between wild-type mice and TLR9^-/-^, cGAS^-/-^, AIM2^-/-^, MyD88^-/-^, STING^gt/gt^, or TLR4^del/del^ mice (Fig. 2B), indicating that the magnitude of nAb responses to the 9vHPV vaccine does not depend on DNA sensing pathways or other pathways tested. Furthermore, as before, male and female mice of all strains elicited similar nAb titers to the 9vHPV vaccine (Fig. S2).

Given that we detected HPV18 L1 DNA in all lots of 9vHPV vaccine tested, including the ones used in mice, but not HPV16 L1 DNA, we also evaluated whether peak HPV18 nAb titers were higher in magnitude than HPV16 nAb titers in any mouse strain tested (Fig. S3). We found no difference between HPV16 and HPV18 nAb titers at week 16 for any mouse strain that received 9vHPV vaccine. However, note that the lack of HPV16 L1 DNA detection in Table 1 does not necessarily mean that HPV16 L1 DNA is not present in 9vHPV vaccine; it simply means we did not detect it with our methods.

### Testing whether long-term 9vHPV vaccine-elicited antibody responses depend on DNA sensing pathways

**Fig 3:**
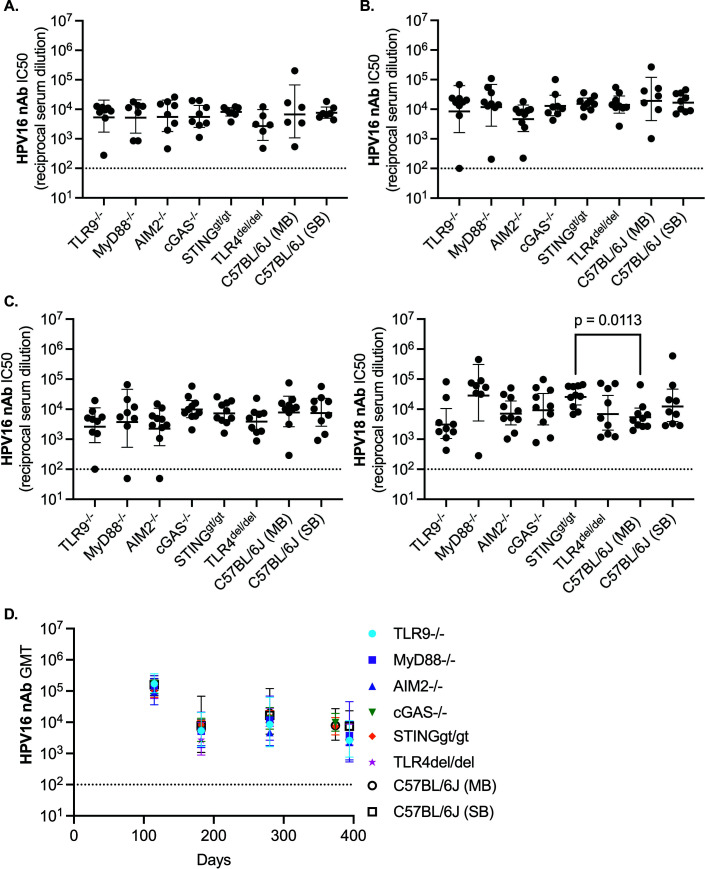
The durability of nAb responses to the 9vHPV vaccine also does not depend on DNA sensing pathways. (**A and B**) Serum HPV16 nAb titers in mice that received 9vHPV, approximately 6 months after the first 9vHPV vaccine dose at week 26 (A; *n* = 6–8 per group), or approximately 9 months after the first 9vHPV vaccine dose at week 40 (B; *n* = 7–10 per group). (**C**) Serum HPV16 or HPV18 nAb titers approximately 1 year after the first 9vHPV vaccine dose at week 53–56 (*n* = 8–10 per group). Geometric mean with 95% confidence intervals shown, where each point represents the result from an individual mouse; Kruskal-Wallis with Dunn’s post-test comparing similarly housed strains to control. (**D**) Change in geometric mean titers (GMT) with 95% confidence intervals of HPV16 nAb responses over time. Dashed line represents cutoff for neutralizing activity; results below this line were non-neutralizing.

To test whether long-term 9vHPV vaccine-elicited antibody responses depend on DNA sensing pathways, we assessed HPV neutralizing activity in sera collected at week 26, 40, and 53–56 after the first 9vHPV vaccine dose (Fig. 3A through C). HPV16 neutralizing activity was assessed at all time points, but due to limited amounts of serum collected by submandibular (facial vein) bleeding at week 26 and 40, HPV18 neutralizing activity was only assessed at week 53–56 (i.e., Fig. 3C), when more blood was collected by cardiac puncture. We found long-term nAb responses to the 9vHPV vaccine did not depend on DNA sensing pathways, MyD88, or TLR4 (Fig. 3A through D). However, it should be noted that global functional knockout of STING significantly improved HPV18 nAb titers by 4.1-fold at week 53–56 (Fig. 3C). In addition, we found no consistent differences in HPV nAb titers between sexes within a strain or between the same sex of different strains at any of the time points tested (Fig. S4). Moreover, when decay rates of log10 HPV16 nAb titers over time were fitted with a linear regression model for each mouse with results from week 16, 26, 40, and 53–56, we observed no difference in nAb titer decay rates across strains (Fig. S5). These data thus indicate that the durability of nAb responses to the 9vHPV vaccine does not depend on DNA sensing pathways or other pathways tested.

### Assessing whether long-lived, 9vHPV vaccine-elicited bone marrow (BM) plasma cell responses depend on DNA sensing pathways

**Fig 4:**
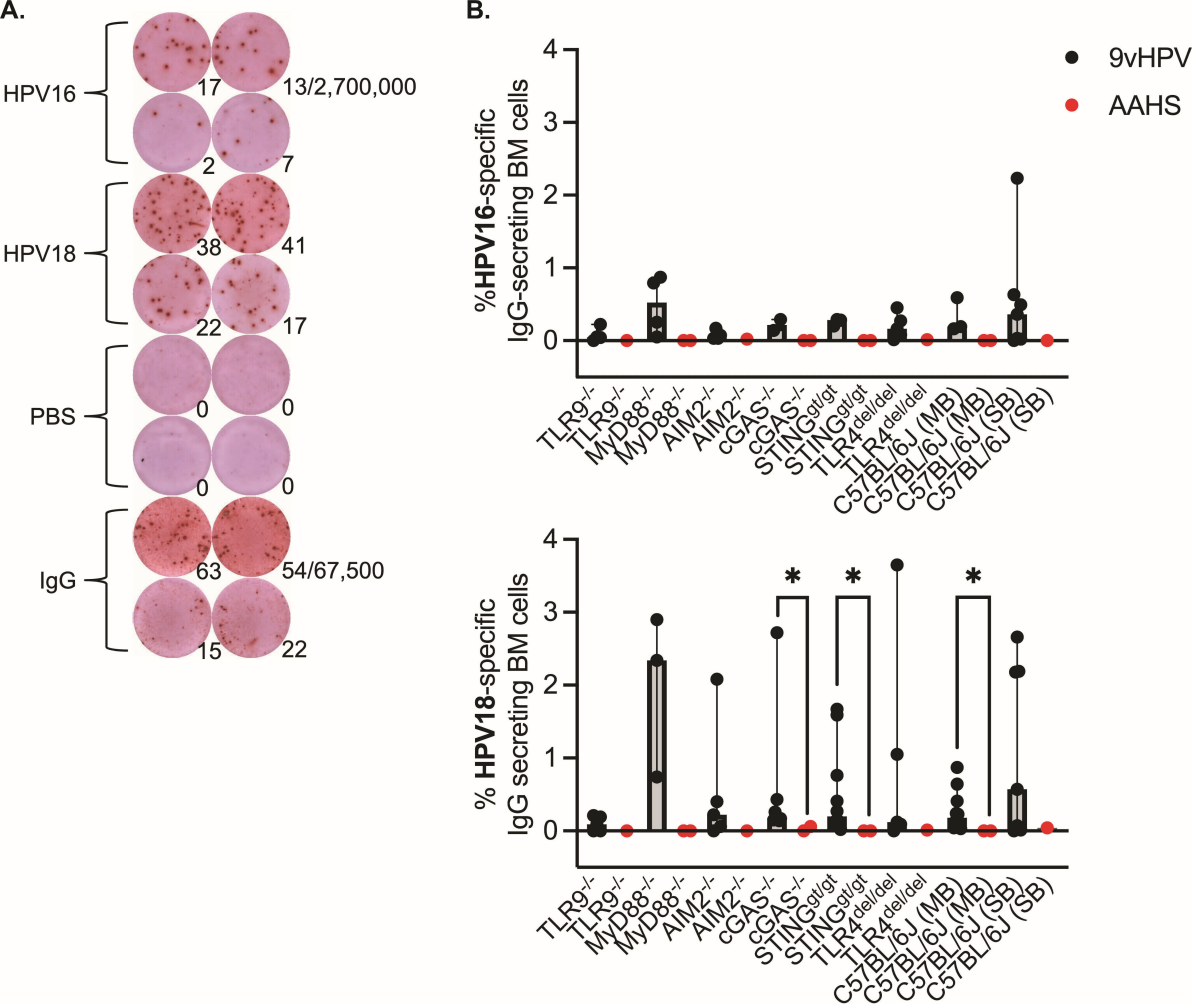
HPV-specific plasma cell responses to the 9vHPV vaccine do not depend on DNA sensing pathways. (**A**) Representative image of ELISPOT plate wells coated with HPV16 or HPV18 VLPs, VLP vehicle (phosphate-buffered saline, PBS), or anti-mouse IgG. Numbers at bottom right of each well indicate spot-forming units (SFUs) per well. Denominators indicate the number of input cells plated in the top dilution row in duplicate (2,700,000 BM cells for VLP or PBS wells; 67,500 BM cells for IgG wells), where the bottom row is a threefold dilution of cells. (**B**) Frequencies of HPV16- or HPV18-specific BM cells of total IgG-secreting BM cells as determined by ELISPOT approximately 1 year after the first 9vHPV vaccine dose at weeks 53–56 (*n* = 2–7 per group HPV16/9vHPV; *n* = 1–2 per group HPV16/AAHS; *n* = 3–10 per group HPV18/9vHPV; *n* = 1–2 per group HPV18/AAHS). Median with 95% confidence intervals shown, where each point represents the result from an individual mouse; Kruskal-Wallis with Dunn’s post-test comparing similarly housed strains to control. Mann-Whitney U-test between 9vHPV and AAHS groups for a given strain (*P = 0.0303–0.0444).

As steady-state antibody levels in blood are replenished by long-lived plasma cells in the BM (51–53), we sought to determine if there were differences in the frequencies of HPV-specific plasma cells in BM approximately a year after the first 9vHPV vaccine dose between wild-type mice and TLR9^-/-^, cGAS^-/-^, AIM2^-/-^, MyD88^-/-^, STING^gt/gt^, or TLR4^del/del^ mice. To do this, we developed an ELISPOT that measured the frequency of HPV16- or HPV18-specific IgG-secreting cells among total IgG-secreting cells in BM (Fig. 4A). To detect HPV16- or HPV18-specific IgG-secreting cells, we coated ELISPOT plates with purified HPV16 or HPV18 VLPs containing L1 and L2, the minor capsid protein (Fig. 4A). We found a significantly lower frequency of HPV18-specific IgG plasma cells in mice that received AAHS versus those receiving 9vHPV vaccine, as expected (Fig. 4B). However, we observed no difference in the frequency of HPV16- or HPV18-specific IgG plasma cells between mouse strains receiving 9vHPV vaccine approximately a year after the first dose (Fig. 4B), further supporting the nAb results that 9vHPV vaccine-elicited B cell responses do not depend on DNA sensing pathways.

### Estimating the relationship between HPV nAb responses in blood and HPV-specific plasma cell responses in BM

**Fig 5:**
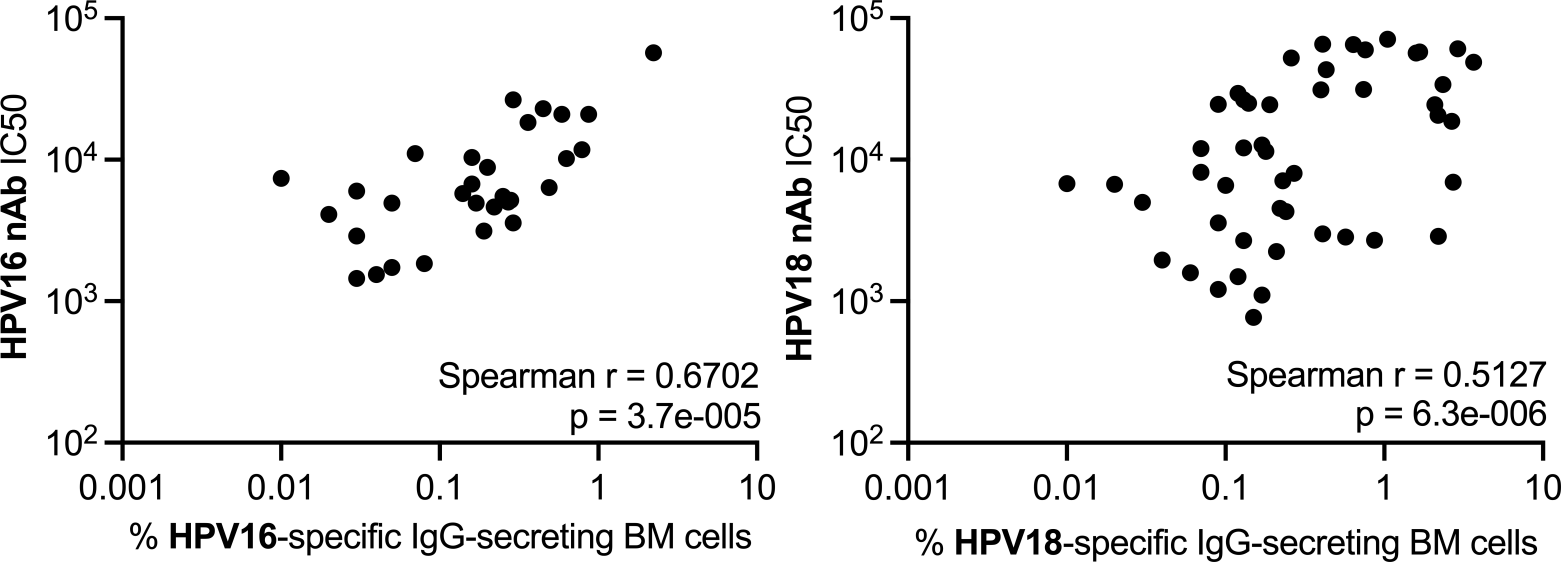
HPV nAb titers correlate with HPV-specific IgG-secreting BM plasma cell frequencies 1 year post-first 9vHPV dose scatter plots of HPV16 or HPV18 nAb titers and frequencies of HPV16- or HPV18-specific IgG-secreting BM cells, respectively, approximately 1 year after the first 9vHPV vaccine dose at weeks 53–56. Each point represents the result from an individual mouse (*n* = 31 for HPV16; *n* = 53 for HPV18); correlation estimated between immune parameters with Spearman coefficients.

To further examine the relationship between nAb titers and BM plasma cell frequencies, we estimated the correlation between HPV16 or HPV18 nAb titers in sera and the frequency of HPV16- or HPV18-specific IgG plasma cells in BM across all mice with paired specimens that received 9vHPV (Fig. 5). We found strong and highly significant positive relationships between these immune parameters for both HPV16 and HPV18 (Fig. 5), as expected given the crucial antibody-secreting role of plasma cells in the BM.

## DISCUSSION

In 2011, the US FDA acknowledged that the 4vHPV vaccine contains residual recombinant HPV L1 DNA as an expected result of the manufacturing process that poses no safety risk (31). As for many protein-based biologics (e.g., subunit vaccines, monoclonal antibodies, etc.), recombinant DNA plasmids are required to express the L1 proteins that are purified for incorporation into the vaccine. Because L1 binds to circular dsDNA, we asked here whether HPV L1 DNA was also present in the updated 9vHPV vaccine and, if so, whether DNA sensing pathways were contributing to the remarkable magnitude and durability of HPV vaccine-elicited antibody responses. Although the total DNA eluted in each dose was below the threshold of detection, we confirmed the presence of low copy numbers of HPV L1 DNA in multiple vaccine lots by quantitative PCR, specifically HPV6 and HPV18 L1 DNA. However, to our surprise, both the magnitude and durability of 9vHPV vaccine-elicited nAb responses in mice did not appear to depend on DNA sensing pathways tested, specifically TLR9, cGAS, or AIM2. Only one cyclic dinucleotide molecule is required to activate each STING dimer to oligomerize (54, 55); AIM2 oligomerizes along a single dsDNA molecule (56); and two CpG molecules activate a TLR9 dimer (57). Thus, while there is theoretically sufficient ligand present in each 9vHPV dose to activate these pathways, it is unclear if the low copy number of L1 DNA we detected would be sufficient to enhance antibody or B cell responses. However, none of these pathways was implicated by our results. We also showed that the magnitude and durability of 9vHPV vaccine-elicited nAb responses do not appear to depend on TLR4 or other TLR pathways that signal through MyD88, including potentially TLR2, which can detect yeast cell wall components (58–60). This is relevant because both the 4vHPV and 9vHPV vaccines are produced in baker’s yeast (*Saccharomyces cerevisiae*) (61, 62).

In contrast to our results, a prior study by Yang et al. found that antibody responses to HPV16 VLPs were lower in MyD88^-/-^ mice and TLR4-hyporesponsive mice compared to wild-type mice (50). Yet, a subsequent study by Thönes et al. did not reproduce the TLR4 result (49). Specifically, Yang et al. found that global knockout of MyD88 eliminated IgG1, IgG2a, IgG2b, and IgG3 responses to HPV16 VLPs (50), in contrast to our finding that global MyD88 knockout had no impact on nAb titers to 9vHPV. There are many differences between this prior study and ours that could account for these disparate results: first, we analyzed serum responses via the gold standard HPV psV neutralization assay that correlates strongly with binding IgG responses in both mice (49) and humans (63), whereas Yang et al. measured serum IgG subclass binding to HPV16 VLPs via ELISA (50); second, we immunized mice via the same route as humans (i.m.), whereas Yang et al. immunized mice intravenously (50); third, our antigen (60 µL 9vHPV) is equivalent to ~7.2 µg HPV16 L1 VLPs expressed from yeast with AAHS adjuvant, whereas the antigen in Yang et al. was 10 µg HPV16 L1 VLPs expressed from baculovirus/insect cells with no adjuvant (50); fourth, we immunized mice on day 0, 28, and 84 and collected blood on day 114 onward, whereas Yang et al. immunized mice on day 0, 7, and 14 and collected blood on day 24 (50); and fifth, the MyD88^-/-^ mice used by Yang et al. were maintained by Prof. Shizuo Akira’s group (50), whereas our mice were from Akira’s group, further backcrossed to C57BL/6J (45), and purchased from The Jackson Laboratory. Yang et al. also observed that global knockout of TLR4 reduced serum IgG3 responses to HPV16 VLPs compared to wild-type mice, as well as serum IgG2a and IgG2b responses (less than IgG3 decrease; no statistical significance shown), but found no change in the predominant IgG1 responses to HPV16 VLPs relative to wild-type mice (50). In this case, the TLR4 mutant mouse strain used also differed from ours (C3H/HeJ mice in the case of Yang et al. [50], and TLR4^del/del^ mice [47, 48] here). When Thönes et al. conducted a similar study in C3H/HeJ (TLR4 mutant) and C3H/HeOuJ (wild-type) mice—the same mice used by Yang et al.—they found no difference in the IgG response to HPV16 VLPs in C3H/HeJ versus C3H/HeOuJ mice (49). In the Thönes et al. study, C3H/HeJ or C3H/HeOuJ mice were immunized subcutaneously with 2.5 or 10 µg HPV16 VLPs produced from baculovirus/insect cells with no adjuvant at day 0 and 14 and with blood collected on day 24 (49). Serum IgG binding to HPV16 VLPs was assessed by capture ELISA (49); so overall, it was more similar to the Yang et al. study. It is not immediately clear what could account for the differences in outcomes in the case of the MyD88^-/-^ mice, but it seems that in the case of the C3H/HeJ mice, the reduction in IgG3, 2a, and 2b responses to VLPs was insufficient to diminish the total IgG response (49), which appeared to be predominantly IgG1 (50). It should also be noted that while the nAb responses to 9vHPV vaccine appear to be durable from approximately month 6 to month 13 in mice—which was not known prior to this study—we do observe a decay from the peak at month 4 to month 6, which, along with the antibody response kinetics following the first, second, and third doses, mimics HPV vaccine-elicited antibody kinetics observed in humans (4, 6, 7, 64, 65). However, murine studies of antibody responses to viral infections reveal antibody responses that do not decay from the peak (53), highlighting crucial differences in the immune responses elicited to viral infections and subunit vaccines in mice that we are keen to explore further.

Limitations of our study include the following: first, a priori, we did not know the L1 DNA sequences utilized to manufacture the vaccines, as they are not publicly available. Therefore, it is possible our PCR primers and probes, which are specific for naturally occurring L1 sequences, are not specific for L1 sequences in the vaccine and thus could not amplify them (e.g., if L1 sequence modifications were made to improve protein expression). Moreover, deep sequencing failed to reveal any HPV DNA sequences. Second, given the number of mice followed long-term, it was not possible to replicate this pilot study. Third, we observed batch effects in neutralization assays, which can be avoided in future studies by testing all specimens from the same mouse together. This was not feasible for this study, and our priority was to determine if there were any differences in nAb titers between strains at any given time point; thus, all specimens from a given time point were tested simultaneously. Fourth, we did not attempt to remove residual DNA in 9vHPV vaccine (e.g., by applying DNase to 9vHPV doses), due to the risk of impacting the structural integrity of the VLPs, which bind to DNA. Fifth, we did not test mice globally deficient in mitochondrial antiviral signaling protein (MAVS) (66), which is a global adaptor for dsRNA retinoic acid-inducible gene I (RIG-I)-like receptors (RLRs) RIG-I and melanoma differentiation-associated protein-5 (MDA5) (67). However, dsDNA viruses, such as herpes, poxviruses, and adenoviruses, can activate RLRs (68). Therefore, this remains a potential future direction.

Other potential future directions include repeating this work in a smaller number of mouse strains (e.g., AIM2^-/-^, MyD88^-/-^, STING^gt/gt^, and wt mice), without nAb testing batch effects, in mice receiving only one or two doses of 9vHPV vaccine. The rationale being that fewer than three HPV vaccine doses are currently recommended by the World Health Organization (69), and differences could become apparent with less saturation of the humoral response. We also aim to examine whether peak memory B cell responses to 9vHPV vaccination differ between mice deficient in DNA sensing pathways relative to wt mice in future work, given that memory B cells and antibody-secreting cells have opposing transcriptional programs (70, 71). This was not feasible here, as we followed a large number of mice for over a year. Depending on the results from these future studies, next steps could include 9vHPV studies in conditional knockout mice (e.g., mice where only B cells or dendritic cells are deficient in DNA sensing pathways).

## MATERIALS AND METHODS

### Quantitative PCR (qPCR)

Published primer and probe sets (42) were used in singleplex reactions for amplification of L1 gene sequences from HPV6, 11, 16, 18, 31, 45, 52, and 58. A new primer-probe set was designed using Primer3 software for HPV33 based on all L1 sequences available in GenBank. Primers were purchased from Integrated DNA Technologies and probes from Biosearch Technologies. All probes but HPV31 were labeled with Cal Fluor Orange 560; HPV31 was labeled with CIV 550. HPV33 primer-probe sequences were evaluated *in silico* using BLAST (National Center for Biotechnology Information) to check exclusivity. These were then evaluated using plasmids containing HPV L1 sequences to identify the set with the most sensitive detection (earliest Ct values). All primers were used at a concentration of 400 nM in the final reaction, and probes were used at 200 nM. Plasmids were diluted to 10^8^, 10^6^, 10^4^, 10^2^, and 10^0^ in molecular-grade water. qPCR was performed using a 20 µL reaction with the Luna Universal Probe qPCR Master Mix (New England Biolabs), using 5 µL of eluted DNA and the following cycling conditions: 95°C for 2 minutes, then 45 cycles of 95°C for 15 seconds and 60°C for 60 seconds. qPCR was run with plasmid dilution series to evaluate the limit of detection. All the primer probe sets had strong curves (data not shown) and detected down to 1 copy/µL of plasmids. A concentration of 10^2^ was selected as a control for the qPCR runs testing eluted 9vHPV vaccine DNA. 9vHPV vaccine doses of 500 µL were split into two 250 µL aliquots and extracted in duplicate using an Apex Kingfisher instrument and the MagMAX viral/pathogen kit. Nucleic acids were eluted in 100 µL of 10 mM Tris-HCl buffer. Five microliters of eluate were then tested in singleplex qPCR for each type. 9vHPV vaccine from three different lots was tested.

### Animal experiments

#### 9vHPV dose titration

Five male and five female C57BL/6J mice (The Jackson Laboratory [JAX] strain # 000664) at 7 weeks of age were assigned to the following dose groups: 10 µL, 20 µL, 30 µL, 60 µL, or 120 µL 9vHPV vaccine (lot 1 in Table 1). 9vHPV doses were administered i.m. at one to three sites (up to 50 µL per site) in the rear thigh muscle at weeks 0, 4, and 11. Blood samples were collected under isoflurane-induced anesthesia by submandibular (facial) vein at weeks 0, 2, 4, 6, 8, 11, and 14 into serum gel separator tubes (Sarstedt 20.1291). Mice were euthanized by American Veterinary Medical Association (AVMA)-approved methods at week 16. Specifically, blood was collected under isoflurane-induced anesthesia by cardiac puncture into serum gel separator tubes (BD Microtainer 365967), and BM was harvested as described below. Sixty microliters of 9vHPV was selected as the optimal dose based on HPV16 and HPV18 neutralization data when analyzed in aggregate for both sexes (i.e., *n* = 10 mice/dose group). All mice were housed in a maximum barrier facility.

#### Assessing the dependency of DNA-sensing pathways on 9vHPV antibody responses

The following mouse strains were used for this experiment: C57BL/6J; AIM2^-/-^ (B6.129P2-Aim2^Gt(CSG445)Byg^/J; JAX strain # 013144 [44]); TLR9^-/-^ (generated through a custom breeding project at JAX with C57BL/6-Tlr9^em1.1Ldm^/J strain # 034449); STING^gt/gt^ (C57BL/6J-Sting1^gt^/J; JAX strain # 017537 [46]); MyD88^-/-^ (B6.129P2(SJL)-Myd88^tm1.1Defr^/J; JAX strain # 009088 [45]); TLR4^del/del^ (B6.B10ScN-Tlr4^lps-del^/JthJ; JAX strain # 007227 [47, 48]); and cGAS^-/-^ (B6(C)-Cgas^tm1d(EUCOMM)Hmgu^/J; JAX strain # 026554 [43]). C57BL/6J, cGAS^-/-^, and STING^gt/gt^ mice were housed in a maximum barrier facility at Emory, whereas C57BL/6J, AIM2^-/-^, TLR9^-/-^, MyD88^-/-^, and TLR4^del/del^ mice were housed in a standard barrier facility. Five male and five female mice of each strain at 5–13 weeks of age were assigned per group, except C57BL/6J mice, which served as controls in each facility. Mice received three doses of 9vHPV vaccine (60 µL per dose; lot 3 in Table 1) or three doses of AAHS (70 µL per dose to yield equivalent AAHS as 60 µL 9vHPV). 9vHPV or AAHS was administered i.m. at 0, 4, and 12 weeks. Blood was collected under isoflurane-induced anesthesia by facial vein at 4, 8, 12, 16, 26, 40, and 53–56 weeks. Mice were euthanized by AVMA-approved methods at 53 weeks (C57BL/6J, cGAS^-/-^, and STING^gt/gt^ mice) or 56 weeks (C57BL/6J, AIM2^-/-^, TLR9^-/-^, MyD88^-/-^, and TLR4^del/del^ mice). On those days, blood was collected under isoflurane-induced anesthesia by cardiac puncture, and BM was harvested.

### Isolation of BM cells

An incision around the upper thigh was made and skin removed. Muscle from the legs was removed with scissors, ensuring not to break the bones in the legs. The hip joint was then cut from the ilium. Legs were placed in ice-cold R10 (Roswell Park Memorial Institute medium [Cytiva SH30255.01 or equivalent], 10% heat-inactivated fetal bovine serum [FBS; R&D Systems #S12450H], 1× penicillin-streptomycin [Cellgro or equivalent], 1× L-glutamine [Gibco or equivalent], 0.1% β-mercaptoethanol [Sigma Aldrich]) with 2 mM EDTA (Quality Biological).

After collection, legs were rinsed in 70% ethanol (Decon Labs) followed by three consecutive rinses in cold sterile 1× PBS (Corning 21-040-CM or equivalent) to remove ethanol from the surface of the legs. Inside a sterile petri dish (VWR), remaining tissue was removed using sterile scalpel and tweezers, and the epiphyses of the bones were removed with scalpel. A 10 mL sterile syringe (Air-Tite) equipped with 25-gauge needle and filled with R10 with 2 mM EDTA was applied to flush the BM from both ends of the bone shaft into a sterile 70 µm cell filter (Falcon) and a 50 mL conical tube (Falcon). The bones were flushed until blanched. The plunger bulb of a sterile 3 mL syringe (Air-Tite) was used to gently press the BM through the filter, and the filter was rinsed with R10 with EDTA. After pelleting cells, red blood cells were lysed with ammonium-chloride-potassium (ACK) lysis buffer (Stemcell Technologies) and quenched with R10 with EDTA. Cells were then pelleted and resuspended in R10 with EDTA prior to counting with a Guava Muse cell analyzer in ViaCount buffer (Cytek).

### Isolation of mouse splenocytes

Mice spleens were homogenized by macerating the spleen on a 100 µm mesh filter (Falcon) with the plunger bulb of a 3 mL syringe. Additional R10 media (without EDTA) was used to flush the spleen through the filter into a conical tube. Cells were pelleted, and red blood cells were lysed with ACK buffer and quenched with R10. The cells were pelleted again, resuspended in R10, filtered through a 70 µm filter into a clean conical tube, and counted with the Guava Muse in ViaCount buffer.

### HPV psV and HPV VLPs

HPV psV were generated using the same procedures as previously described in Scherer et al. (72), except that psV were purified by density ultracentrifugation on 0.7 mL gradients at 41,000 rpm for ~6 hours at 16°C in a SW Ti 41 rotor in 13.2 mL sterile ultracentrifuge tubes (Beckman Coulter C14293). HPV VLPs were generated for BM ELISPOTs in the same manner, except no pYSEAP was used during transfection, and 0.25% benzonase (SigmaAldrich EB263) and 0.25% plasmid-safe DNase (Biosearch Technologies E3101K) were used instead of RNase during maturation. Density gradient fractions containing psV and VLPs were screened by ELISA and psV titered or VLPs quantified by L1 SDS-PAGE densitometry with reference to BSA standards prior to use as described (72).

### HPV psV neutralization assay

The same procedure was used as previously described in Scherer et al. (72) except that cells were seeded into an Edgewell 96-well plate (Thermo Fisher Scientific 167542). The outside troughs of the plate were filled with 3 mL of 1× PBS before incubating at 37°C for 4 hours. Sera were initially screened at 1:100 starting dilution in duplicate in a threefold dilution series. Potently neutralizing sera were diluted further (e.g., 1:2,700 starting dilution) in subsequent experiments prior to analyzing in duplicate.

### BM ELISPOT

Millipore Multiscreen-IP 96-well plates (MSIPS4510) were coated with antigens 24 hours prior to use. ELISPOT plates were washed according to manufacturer recommendations with 35% ethanol, deionized water, and 1× PBS. Plates were coated with donkey anti-mouse IgG antibody (Jackson ImmunoResearch 715-005-150) at 10 µg/mL in 1× PBS, 1× PBS, or HPV16 or HPV18 VLPs at 10 µg/mL 1× PBS. Depending on cell yield, each sample was tested in duplicate for each antigen. Plates were stored at 4°C until use.

After 24 hours, coated plates were washed four times with PBS-T (0.05% (vol/vol) Tween-20 (Fisher Scientific or equivalent) in 1× PBS) and blocked with R10 with 2 mM EDTA at 37°C for 1–2 hours. After blocking, R10 with EDTA was added to all wells. Two to four million BM cells were then added to the first well of HPV VLPs or PBS wells, and 50,000–100,000 BM cells were added to the first well of the IgG wells. Then one threefold serial cellular dilution was performed and plates incubated at 37°C for approximately 18 hours.

After ~18 hours, plates were washed with 1× PBS and then with PBS-T. Biotinylated anti-mouse IgG antibody (Jackson ImmunoResearch 115-065-071) in 50% glycerol and 50% water was diluted to 1/500 in PBS-T with 2% (wt/vol) HI-FBS and incubated in wells for 2 hours at room temperature. Plates were then washed with PBS-T and horseradish peroxidase-conjugated Avidin D (Vector Laboratories A-2014) diluted 1/1,000 in PBS-T with 2% HI-FBS was added to each well for 1 hour at room temperature. Plates were then washed with PBS-T and then with 1× PBS. AEC substrate (BD Biosciences 551951) was added to each well, and plates were placed in the dark for approximately 5 minutes. Plates were then rinsed in cold tap water, and the backing removed and rinsed. Plates were stored protected from light to dry and read within 2 working days.

### Sequencing

The total nucleic acid in each eluate was quantified using the Qubit dsDNA High Sensitivity Assay (Thermo Fisher Scientific) following the manufacturer’s protocol. Unique dual-indexed libraries were generated from each DNA eluate using the Nextera XT Kit (Illumina) according to the manufacturer’s instructions. A nuclease-free water sample was included as a negative control. The libraries were quantified using the KAPA Universal Kit (Roche) and normalized and pooled at an equimolar concentration. Sequencing was performed on a MiSeq instrument (Illumina).

### Analysis

#### Neutralization assay

Absorbance was read at 405 nm. The average absorbance of background (cells-only wells) was subtracted from each psV or psV+sample well absorbance prior to averaging signal in psV-only wells and determining percent neutralization for each sample:


$$\left[\frac{\left({\text{ Mean }}_{\text{background-subtracted psV signal }}\right)-\left({\text{ Mean }}_{\text{background-subtracted psV+sample signal }}\right)}{{\text{ Mean }}_{\text{background-subtracted psV signal }}}\right]x100$$

If the maximum average percent neutralization was less than 50%, the sample was considered non-neutralizing and IC50 reported as half the reciprocal of the starting dilution (i.e., 50). If the maximum average percent neutralization was 50%, or just above 50%, for the starting dilution only, the sample was considered neutralizing and IC50 reported as the reciprocal of the starting dilution (i.e., 100). Otherwise, IC50 values were determined by fitting percent neutralization data curves using four-parameter nonlinear regression (Prism v.10).

#### ELISPOT

Plates were scanned using Autocenter on a CTL ImmunoSpot S6 Universal Analyzer. Scans were counted using ImmunoSpot 5.3.22 software, Basic Count. SFU per well were normalized to the number of BM cells added per well at each cellular dilution. SFU per well were averaged across all cellular dilutions of a given sample-antigen pair and then background-corrected (i.e., the average SFU per well of corresponding cellular dilutions in PBS wells were subtracted). The frequency of HPV16- or HPV18-specific plasma cells was reported as a percentage of IgG-secreting plasma cells.

#### Sequencing

Sequencing reads underwent metagenomic classification using viral-ngs (73).

## Data Availability

All data are included in the article. No new accession numbers, microarray data, protein structures, or gene expression data are associated with this study.

## SUPPLEMENTAL MATERIAL

Supplemental figures jvi.02150-25-s0001.pdf Figures S1 to S5.

ASM does not own the copyrights to Supplemental Material that may be linked to, or accessed through, an article. The authors have granted ASM a non-exclusive, world-wide license to publish the Supplemental Material files. Please contact the corresponding author directly for reuse.

## References

[1] Plotkin SA. 2010. Correlates of protection induced by vaccination. Clin Vaccine Immunol 17:1055–1065. doi:10.1128/CVI.00131-10 20463105 PMC2897268

[2] Plotkin SA. 2020. Updates on immunologic correlates of vaccine-induced protection. Vaccine 38:2250–2257. doi:10.1016/j.vaccine.2019.10.046 31767462

[3] Kjaer SK, Nygård M, Sundström K, Dillner J, Tryggvadottir L, Munk C, Berger S, Enerly E, Hortlund M, Ágústsson ÁI, Bjelkenkrantz K, Fridrich K, Guðmundsdóttir I, Sørbye SW, Bautista O, Group T, Luxembourg A, Marshall JB, Radley D, Yang YS, Badshah C, Saah A. 2020. Final analysis of a 14-year long-term follow-up study of the effectiveness and immunogenicity of the quadrivalent human papillomavirus vaccine in women from four nordic countries. EClinicalMedicine 23:100401. doi:10.1016/j.eclinm.2020.100401 32637895 PMC7329692

[4] Donken R, Dobson SRM, Marty KD, Cook D, Sauvageau C, Gilca V, Dionne M, McNeil S, Krajden M, Money D, Kellner J, Scheifele DW, Kollmann T, Bettinger JA, Liu S, Singer J, Naus M, Sadarangani M, Ogilvie GS. 2020. Immunogenicity of 2 and 3 doses of the quadrivalent human papillomavirus vaccine up to 120 months postvaccination: follow-up of a randomized clinical trial. Clin Infect Dis 71:1022–1029. doi:10.1093/cid/ciz887 31617568 PMC7428395

[5] Artemchuk H, Eriksson T, Poljak M, Surcel HM, Dillner J, Lehtinen M, Faust H. 2019. Long-term antibody response to human papillomavirus vaccines: up to 12 years of follow-up in the finnish maternity cohort. J Infect Dis 219:582–589. doi:10.1093/infdis/jiy545 30239832

[6] Olsson S-E, Restrepo JA, Reina JC, Pitisuttithum P, Ulied A, Varman M, Van Damme P, Moreira ED Jr, Ferris D, Block S, Bautista O, Gallagher N, McCauley J, Luxembourg A. 2020. Long-term immunogenicity, effectiveness, and safety of nine-valent human papillomavirus vaccine in girls and boys 9 to 15 years of age: interim analysis after 8 years of follow-up. Papillomavirus Res 10:100203. doi:10.1016/j.pvr.2020.100203 32659510 PMC7396911

[7] Godi A, Panwar K, Haque M, Cocuzza CE, Andrews N, Southern J, Turner P, Miller E, Beddows S. 2019. Durability of the neutralizing antibody response to vaccine and non-vaccine HPV types 7 years following immunization with either Cervarix or Gardasil vaccine. Vaccine 37:2455–2462. doi:10.1016/j.vaccine.2019.03.052 30926298

[8] Slifka MK, Amanna IJ. 2019. Role of multivalency and antigenic threshold in generating protective antibody responses. Front Immunol 10:956. doi:10.3389/fimmu.2019.00956 31118935 PMC6504826

[9] Krammer F. 2019. The human antibody response to influenza A virus infection and vaccination. Nat Rev Immunol 19:383–397. doi:10.1038/s41577-019-0143-6 30837674

[10] Pool V, Tomovici A, Johnson DR, Greenberg DP, Decker MD. 2018. Humoral immunity 10 years after booster immunization with an adolescent and adult formulation combined tetanus, diphtheria, and 5-component acellular pertussis vaccine in the USA. Vaccine 36:2282–2287. doi:10.1016/j.vaccine.2018.03.029 29573876

[11] Amanna IJ, Carlson NE, Slifka MK. 2007. Duration of humoral immunity to common viral and vaccine antigens. N Engl J Med 357:1903–1915. doi:10.1056/NEJMoa066092 17989383

[12] Mendy M, Peterson I, Hossin S, Peto T, Jobarteh ML, Jeng-Barry A, Sidibeh M, Jatta A, Moore SE, Hall AJ, Whittle H. 2013. Observational study of vaccine efficacy 24 years after the start of hepatitis B vaccination in two Gambian villages: no need for a booster dose. PLoS One 8:e58029. doi:10.1371/journal.pone.0058029 23533578 PMC3606345

[13] Corey L, Gilbert PB, Tomaras GD, Haynes BF, Pantaleo G, Fauci AS. 2015. Immune correlates of vaccine protection against HIV-1 acquisition. Sci Transl Med 7:310rv7. doi:10.1126/scitranslmed.aac7732 PMC4751141 26491081

[14] Ochiai RL, Khan MI, Soofi SB, Sur D, Kanungo S, You YA, Habib MA, Sahito SM, Manna B, Dutta S, Acosta CJ, Ali M, Bhattacharya SK, Bhutta ZA, Clemens JD. 2014. Immune responses to Vi capsular polysaccharide typhoid vaccine in children 2 to 16 years old in Karachi, Pakistan, and Kolkata, India. Clin Vaccine Immunol 21:661–666. doi:10.1128/CVI.00791-13 24599532 PMC4018880

[15] Keitel WA, Bond NL, Zahradnik JM, Cramton TA, Robbins JB. 1994. Clinical and serological responses following primary and booster immunization with Salmonella Typhi Vi capsular polysaccharide vaccines. Vaccine 12:195–199. doi:10.1016/0264-410X(94)90194-5 8165850

[16] Froeschle JE, Decker MD. 2010. Duration of Vi antibodies in participants vaccinated with Typhim Vi (Typhoid Vi polysaccharide vaccine) in an area not endemic for typhoid fever. Vaccine 28:1451–1453. doi:10.1016/j.vaccine.2009.11.051 20003920

[17] Falsey AR, Frenck RW Jr, Walsh EE, Kitchin N, Absalon J, Gurtman A, Lockhart S, Bailey R, Swanson KA, Xu X, Koury K, Kalina W, Cooper D, Zou J, Xie X, Xia H, Türeci Ö, Lagkadinou E, Tompkins KR, Shi P-Y, Jansen KU, Şahin U, Dormitzer PR, Gruber WC. 2021. SARS-CoV-2 neutralization with BNT162b2 vaccine dose 3. N Engl J Med 385:1627–1629. doi:10.1056/NEJMc2113468 34525276 PMC8461567

[18] Joura EA, Giuliano AR, Iversen O-E, Bouchard C, Mao C, Mehlsen J, Moreira ED Jr, Ngan Y, Petersen LK, Lazcano-Ponce E, et al.. 2015. A 9-valent HPV vaccine against infection and intraepithelial neoplasia in women. N Engl J Med 372:711–723. doi:10.1056/NEJMoa1405044 25693011

[19] de Sanjose S, Quint WG, Alemany L, Geraets DT, Klaustermeier JE, Lloveras B, Tous S, Felix A, Bravo LE, Shin H-R, et al.. 2010. Human papillomavirus genotype attribution in invasive cervical cancer: a retrospective cross-sectional worldwide study. Lancet Oncol 11:1048–1056. doi:10.1016/S1470-2045(10)70230-8 20952254

[20] Srodon M, Stoler MH, Baber GB, Kurman RJ. 2006. The distribution of low and high-risk HPV types in vulvar and vaginal intraepithelial neoplasia (VIN and VaIN). Am J Surg Pathol 30:1513–1518. doi:10.1097/01.pas.0000213291.96401.48 17122506

[21] Hoots BE, Palefsky JM, Pimenta JM, Smith JS. 2009. Human papillomavirus type distribution in anal cancer and anal intraepithelial lesions. Int J Cancer 124:2375–2383. doi:10.1002/ijc.24215 19189402

[22] Chaturvedi AK. 2010. Beyond cervical cancer: burden of other HPV-related cancers among men and women. J Adolesc Health 46:S20–S26. doi:10.1016/j.jadohealth.2010.01.016 20307840

[23] Baker TS, Newcomb WW, Olson NH, Cowsert LM, Olson C, Brown JC. 1991. Structures of bovine and human papillomaviruses. Analysis by cryoelectron microscopy and three-dimensional image reconstruction. Biophys J 60:1445–1456. doi:10.1016/S0006-3495(91)82181-6 1663794 PMC1260204

[24] Chen XS, Garcea RL, Goldberg I, Casini G, Harrison SC. 2000. Structure of small virus-like particles assembled from the L1 protein of human papillomavirus 16. Mol Cell 5:557–567. doi:10.1016/s1097-2765(00)80449-9 10882140

[25] Li Z, Yan X, Yu H, Wang D, Song S, Li Y, He M, Hong Q, Zheng Q, Zhao Q, Gu Y, Zhang J, Janssen MEW, Cardone G, Olson NH, Baker TS, Li S, Xia N. 2016. The C-terminal arm of the human papillomavirus major capsid protein is immunogenic and involved in virus-host interaction. Structure 24:874–885. doi:10.1016/j.str.2016.04.008 27276427 PMC5595370

[26] Schäfer F, Florin L, Sapp M. 2002. DNA binding of L1 is required for human papillomavirus morphogenesis in vivo. Virology 295:172–181. doi:10.1006/viro.2002.1361 12033775

[27] DiGiuseppe S, Bienkowska-Haba M, Guion LGM, Keiffer TR, Sapp M. 2017. Human papillomavirus major capsid protein L1 remains associated with the incoming viral genome throughout the entry process. J Virol 91:e00537-17. doi:10.1128/JVI.00537-17 28566382 PMC5533910

[28] Doorbar J, Quint W, Banks L, Bravo IG, Stoler M, Broker TR, Stanley MA. 2012. The biology and life-cycle of human papillomaviruses. Vaccine 30 Suppl 5:F55–F70. doi:10.1016/j.vaccine.2012.06.083 23199966

[29] Schiffman M, Doorbar J, Wentzensen N, de Sanjosé S, Fakhry C, Monk BJ, Stanley MA, Franceschi S. 2016. Carcinogenic human papillomavirus infection. Nat Rev Dis Primers 2:16086. doi:10.1038/nrdp.2016.86 27905473

[30] Unckell F, Streeck RE, Sapp M. 1997. Generation and neutralization of pseudovirions of human papillomavirus type 33. J Virol 71:2934–2939. doi:10.1128/JVI.71.4.2934-2939.1997 9060652 PMC191421

[31] FDA. 2011. FDA information on Gardasil – presence of DNA fragments expected, no safety risk. https://downloads.regulations.gov/FDA-2020-P-2225-0011/attachment_16.pdf.

[32] Hornung V, Latz E. 2010. Intracellular DNA recognition. Nat Rev Immunol 10:123–130. doi:10.1038/nri2690 20098460

[33] Motwani M, Pesiridis S, Fitzgerald KA. 2019. DNA sensing by the cGAS-STING pathway in health and disease. Nat Rev Genet 20:657–674. doi:10.1038/s41576-019-0151-1 31358977

[34] Hanson MC, Crespo MP, Abraham W, Moynihan KD, Szeto GL, Chen SH, Melo MB, Mueller S, Irvine DJ. 2015. Nanoparticulate STING agonists are potent lymph node-targeted vaccine adjuvants. J Clin Invest 125:2532–2546. doi:10.1172/JCI79915 25938786 PMC4497758

[35] Junkins RD, Gallovic MD, Johnson BM, Collier MA, Watkins-Schulz R, Cheng N, David CN, McGee CE, Sempowski GD, Shterev I, McKinnon K, Bachelder EM, Ainslie KM, Ting JP-Y. 2018. A robust microparticle platform for a STING-targeted adjuvant that enhances both humoral and cellular immunity during vaccination. J Control Release 270:1–13. doi:10.1016/j.jconrel.2017.11.030 29170142 PMC5808851

[36] Rookhuizen DC, DeFranco AL. 2014. Toll-like receptor 9 signaling acts on multiple elements of the germinal center to enhance antibody responses. Proc Natl Acad Sci USA 111:E3224–E3233. doi:10.1073/pnas.1323985111 25053813 PMC4128120

[37] Demento SL, Bonafé N, Cui W, Kaech SM, Caplan MJ, Fikrig E, Ledizet M, Fahmy TM. 2010. TLR9-targeted biodegradable nanoparticles as immunization vectors protect against West Nile encephalitis. J Immunol 185:2989–2997. doi:10.4049/jimmunol.1000768 20660705 PMC3753007

[38] Jackson S, Lentino J, Kopp J, Murray L, Ellison W, Rhee M, Shockey G, Akella L, Erby K, Heyward WL, Janssen RS, Group HBVS. 2018. Immunogenicity of a two-dose investigational hepatitis B vaccine, HBsAg-1018, using a toll-like receptor 9 agonist adjuvant compared with a licensed hepatitis B vaccine in adults. Vaccine 36:668–674. doi:10.1016/j.vaccine.2017.12.038 29289383

[39] Halperin SA, Ward B, Cooper C, Predy G, Diaz-Mitoma F, Dionne M, Embree J, McGeer A, Zickler P, Moltz KH, Martz R, Meyer I, McNeil S, Langley JM, Martins E, Heyward WL, Martin JT. 2012. Comparison of safety and immunogenicity of two doses of investigational hepatitis B virus surface antigen co-administered with an immunostimulatory phosphorothioate oligodeoxyribonucleotide and three doses of a licensed hepatitis B vaccine in healthy adults 18-55 years of age. Vaccine 30:2556–2563. doi:10.1016/j.vaccine.2012.01.087 22326642

[40] Stahl-Hennig C, Eisenblätter M, Jasny E, Rzehak T, Tenner-Racz K, Trumpfheller C, Salazar AM, Uberla K, Nieto K, Kleinschmidt J, Schulte R, Gissmann L, Müller M, Sacher A, Racz P, Steinman RM, Uguccioni M, Ignatius R. 2009. Synthetic double-stranded RNAs are adjuvants for the induction of T helper 1 and humoral immune responses to human papillomavirus in rhesus macaques. PLoS Pathog 5:e1000373. doi:10.1371/journal.ppat.1000373 19360120 PMC2660151

[41] Fernandez AF, Rosales C, Lopez-Nieva P, Graña O, Ballestar E, Ropero S, Espada J, Melo SA, Lujambio A, Fraga MF, et al.. 2009. The dynamic DNA methylomes of double-stranded DNA viruses associated with human cancer. Genome Res 19:438–451. doi:10.1101/gr.083550.108 19208682 PMC2661803

[42] Roberts CC, Swoyer R, Bryan JT, Taddeo FJ. 2011. Comparison of real-time multiplex human papillomavirus (HPV) PCR assays with the linear array HPV genotyping PCR assay and influence of DNA extraction method on HPV detection. J Clin Microbiol 49:1899–1906. doi:10.1128/JCM.00235-10 21346041 PMC3122643

[43] Schoggins JW, MacDuff DA, Imanaka N, Gainey MD, Shrestha B, Eitson JL, Mar KB, Richardson RB, Ratushny AV, Litvak V, Dabelic R, Manicassamy B, Aitchison JD, Aderem A, Elliott RM, García-Sastre A, Racaniello V, Snijder EJ, Yokoyama WM, Diamond MS, Virgin HW, Rice CM. 2014. Pan-viral specificity of IFN-induced genes reveals new roles for cGAS in innate immunity. Nature 505:691–695. doi:10.1038/nature12862 24284630 PMC4077721

[44] Rathinam VAK, Jiang Z, Waggoner SN, Sharma S, Cole LE, Waggoner L, Vanaja SK, Monks BG, Ganesan S, Latz E, Hornung V, Vogel SN, Szomolanyi-Tsuda E, Fitzgerald KA. 2010. The AIM2 inflammasome is essential for host defense against cytosolic bacteria and DNA viruses. Nat Immunol 11:395–402. doi:10.1038/ni.1864 20351692 PMC2887480

[45] Hou B, Reizis B, DeFranco AL. 2008. Toll-like receptors activate innate and adaptive immunity by using dendritic cell-intrinsic and -extrinsic mechanisms. Immunity 29:272–282. doi:10.1016/j.immuni.2008.05.016 18656388 PMC2847796

[46] Sauer J-D, Sotelo-Troha K, von Moltke J, Monroe KM, Rae CS, Brubaker SW, Hyodo M, Hayakawa Y, Woodward JJ, Portnoy DA, Vance RE. 2011. The N-ethyl-N-nitrosourea-induced Goldenticket mouse mutant reveals an essential function of Sting in the in vivo interferon response to Listeria monocytogenes and cyclic dinucleotides. Infect Immun 79:688–694. doi:10.1128/IAI.00999-10 21098106 PMC3028833

[47] Vogel SN, Hansen CT, Rosenstreich DL. 1979. Characterization of a congenitally LPS-resistant, athymic mouse strain. J Immunol 122:619–622. doi:10.4049/jimmunol.122.2.619 368244

[48] Poltorak A, He X, Smirnova I, Liu MY, Van Huffel C, Du X, Birdwell D, Alejos E, Silva M, Galanos C, Freudenberg M, Ricciardi-Castagnoli P, Layton B, Beutler B. 1998. Defective LPS signaling in C3H/HeJ and C57BL/10ScCr mice: mutations in Tlr4 gene. Science 282:2085–2088. doi:10.1126/science.282.5396.2085 9851930

[49] Thönes N, Herreiner A, Schädlich L, Piuko K, Müller M. 2008. A direct comparison of human papillomavirus type 16 L1 particles reveals a lower immunogenicity of capsomeres than viruslike particles with respect to the induced antibody response. J Virol 82:5472–5485. doi:10.1128/JVI.02482-07 18385253 PMC2395182

[50] Yang R, Murillo FM, Delannoy MJ, Blosser RL, Yutzy WH IV, Uematsu S, Takeda K, Akira S, Viscidi RP, Roden RBS. 2005. B lymphocyte activation by human papillomavirus-like particles directly induces Ig class switch recombination via TLR4-MyD88. J Immunol 174:7912–7919. doi:10.4049/jimmunol.174.12.7912 15944297

[51] Slifka MK, Antia R, Whitmire JK, Ahmed R. 1998. Humoral immunity due to long-lived plasma cells. Immunity 8:363–372. doi:10.1016/s1074-7613(00)80541-5 9529153

[52] Hammarlund E, Thomas A, Amanna IJ, Holden LA, Slayden OD, Park B, Gao L, Slifka MK. 2017. Plasma cell survival in the absence of B cell memory. Nat Commun 8:1781. doi:10.1038/s41467-017-01901-w 29176567 PMC5701209

[53] Langley WA, Wieland A, Ahmed H, Rasheed MAU, Davis CW, Sewatanon J, Mueller SN, Shlomchik MJ, Zarnitsyna VI, Antia R, Ahmed R. 2022. Persistence of virus-specific antibody after depletion of memory B cells. J Virol 96:e0002622. doi:10.1128/jvi.00026-22 35404084 PMC9093124

[54] Shang G, Zhang C, Chen ZJ, Bai X-C, Zhang X. 2019. Cryo-EM structures of STING reveal its mechanism of activation by cyclic GMP-AMP. Nature 567:389–393. doi:10.1038/s41586-019-0998-5 30842659 PMC6859894

[55] Zhang B, Xu P, Ablasser A. 2025. Regulation of the cGAS-STING pathway. Annu Rev Immunol 43:667–692. doi:10.1146/annurev-immunol-101721-032910 40085836

[56] Sharma M, de Alba E. 2023. Assembly mechanism of the inflammasome sensor AIM2 revealed by single molecule analysis. Nat Commun 14:7957. doi:10.1038/s41467-023-43691-4 38042863 PMC10693601

[57] Ohto U, Shibata T, Tanji H, Ishida H, Krayukhina E, Uchiyama S, Miyake K, Shimizu T. 2015. Structural basis of CpG and inhibitory DNA recognition by Toll-like receptor 9. Nature 520:702–705. doi:10.1038/nature14138 25686612

[58] Silva RL, Lopes AH, Becerra A, Fonseca MM, Maganin A, Saraiva ALL, Cunha FQ, Alves-Filho JC, Zamboni DS, Cunha TM. 2024. Molecular mechanisms of zymosan-induced inflammasome activation in macrophages. Cell Signal 124:111418. doi:10.1016/j.cellsig.2024.111418 39304096

[59] Rogers NC, Slack EC, Edwards AD, Nolte MA, Schulz O, Schweighoffer E, Williams DL, Gordon S, Tybulewicz VL, Brown GD, Reis e Sousa C. 2005. Syk-dependent cytokine induction by Dectin-1 reveals a novel pattern recognition pathway for C type lectins. Immunity 22:507–517. doi:10.1016/j.immuni.2005.03.004 15845454

[60] Dillon S, Agrawal S, Banerjee K, Letterio J, Denning TL, Oswald-Richter K, Kasprowicz DJ, Kellar K, Pare J, van Dyke T, Ziegler S, Unutmaz D, Pulendran B. 2006. Yeast zymosan, a stimulus for TLR2 and dectin-1, induces regulatory antigen-presenting cells and immunological tolerance. J Clin Invest 116:916–928. doi:10.1172/JCI27203 16543948 PMC1401484

[61] FDA. 2025. Gardasil Package Insert. Available from: https://www.fda.gov/vaccines-blood-biologics/vaccines/gardasil

[62] Gardasil-9 package insert FDA. 2025. Available from: https://www.fda.gov/vaccines-blood-biologics/vaccines/gardasil-9

[63] Tsang SH, Basu P, Bender N, Herrero R, Kemp TJ, Kreimer AR, Müller M, Panicker G, Pawlita M, Pinto LA, Sampson JN, Sankaranarayanan R, Schussler J, Sehr P, Sierra MS, Unger ER, Waterboer T, Hildesheim A, Costa Rica Vaccine Trial (CVT) Group. 2020. Evaluation of serological assays to monitor antibody responses to single-dose HPV vaccines. Vaccine 38:5997–6006. doi:10.1016/j.vaccine.2020.07.017 32713678 PMC7429278

[64] Olsson S-E, Villa LL, Costa RLR, Petta CA, Andrade RP, Malm C, Iversen O-E, Høye J, Steinwall M, Riis-Johannessen G, Andersson-Ellstrom A, Elfgren K, von Krogh G, Lehtinen M, Paavonen J, Tamms GM, Giacoletti K, Lupinacci L, Esser MT, Vuocolo SC, Saah AJ, Barr E. 2007. Induction of immune memory following administration of a prophylactic quadrivalent human papillomavirus (HPV) types 6/11/16/18 L1 virus-like particle (VLP) vaccine. Vaccine 25:4931–4939. doi:10.1016/j.vaccine.2007.03.049 17499406

[65] Einstein MH, Takacs P, Chatterjee A, Sperling RS, Chakhtoura N, Blatter MM, Lalezari J, David MP, Lin L, Struyf F, Dubin G, Group HPVS. 2014. Comparison of long-term immunogenicity and safety of human papillomavirus (HPV)-16/18 AS04-adjuvanted vaccine and HPV-6/11/16/18 vaccine in healthy women aged 18-45 years: end-of-study analysis of a Phase III randomized trial. Hum Vaccin Immunother 10:3435–3445. doi:10.4161/hv.36121 25483701 PMC4514070

[66] Sun Q, Sun L, Liu HH, Chen X, Seth RB, Forman J, Chen ZJ. 2006. The specific and essential role of MAVS in antiviral innate immune responses. Immunity 24:633–642. doi:10.1016/j.immuni.2006.04.004 16713980

[67] Chen YG, Hur S. 2022. Cellular origins of dsRNA, their recognition and consequences. Nat Rev Mol Cell Biol 23:286–301. doi:10.1038/s41580-021-00430-1 34815573 PMC8969093

[68] Rehwinkel J, Gack MU. 2020. RIG-I-like receptors: their regulation and roles in RNA sensing. Nat Rev Immunol 20:537–551. doi:10.1038/s41577-020-0288-3 32203325 PMC7094958

[69] WHO. 2022. Human papillomavirus vaccines: WHO position paper (2022 update). World Health Organization, Weekly Epidemiological Record. Available from: https://www.who.int/publications/i/item/who-wer9750-645-672

[70] Laidlaw BJ, Cyster JG. 2021. Transcriptional regulation of memory B cell differentiation. Nat Rev Immunol 21:209–220. doi:10.1038/s41577-020-00446-2 33024284 PMC7538181

[71] Victora GD, Nussenzweig MC. 2022. Germinal centers. Annu Rev Immunol 40:413–442. doi:10.1146/annurev-immunol-120419-022408 35113731

[72] Scherer EM, Smith RA, Simonich CA, Niyonzima N, Carter JJ, Galloway DA. 2014. Characteristics of memory B cells elicited by a highly efficacious HPV vaccine in subjects with no pre-existing immunity. PLoS Pathog 10:e1004461. doi:10.1371/journal.ppat.1004461 25330199 PMC4199765

[73] Park DJ, Dudas G, Wohl S, Goba A, Whitmer SLM, Andersen KG, Sealfon RS, Ladner JT, Kugelman JR, Matranga CB, et al.. 2015. Ebola virus epidemiology, transmission, and evolution during seven months in Sierra Leone. Cell 161:1516–1526. doi:10.1016/j.cell.2015.06.007 26091036 PMC4503805

